# Acute Cytotoxic Effects on Morphology and Mechanical Behavior in MCF-7 Induced by TiO_2_NPs Exposure

**DOI:** 10.3390/ijms20143594

**Published:** 2019-07-23

**Authors:** Mariafrancesca Cascione, Valeria De Matteis, Giacomo Mandriota, Stefano Leporatti, Rosaria Rinaldi

**Affiliations:** 1 Department of Mathematics and Physics “E. De Giorgi”, University of Salento, Via Monteroni, 73100 Lecce, Italy; 2 IIT—Italian Institute of Technology, via Morego 30, 16163 Genova, Italy; 3 CNR Nanotec—Institute of Nanotechnology, 73100 Lecce, Italy

**Keywords:** biomechanics, scanning force microscopy, cytotoxicity

## Abstract

The side effects induced by nanoparticle exposure at a cellular level are one of the priority research topics due to the steady increase in the use of nanoparticles (NPs). Recently, the focus on cellular morphology and mechanical behavior is gaining relevance in order to fully understand the cytotoxic mechanisms. In this regard, we have evaluated the morphomechanical alteration in human breast adenocarcinoma cell line (MCF-7) exposed to TiO_2_NPs at two different concentrations (25 and 50 µg/mL) and two time points (24 and 48 h). By using confocal and atomic force microscopy, we demonstrated that TiO_2_NP exposure induces significant alterations in cellular membrane elasticity, due to actin proteins rearrangement in cytoskeleton, as calculated in correspondence to nuclear and cytoplasmic compartments. In this work, we have emphasized the alteration in mechanical properties of the cellular membrane, induced by nanoparticle exposure.

## 1. Introduction

In the last two decades, the advances in nanotechnology field led to the rapid development of novel applications of engineered nanoparticles (ENPs) ranging from industrial [1] to commercial and biomedical [2,3]. The growing production and applications of nanoparticles (NPs) inevitably entail a potential hazard to environment and living organisms. Nowadays, the massive use of ENPs urges the evaluation of their potential toxicity and, in this perspective, the in vitro studies represent a critical point to assess the nanostructures’ impact at a cellular level before in vivo investigations.

Among all the nanomaterials, titanium dioxide NPs (TiO_2_NPs) are the subject of great attention from the scientific community, mainly for their broad spectrum of applications in several commercial goods, such as cosmetics, plastics, paper and food [4,5,6,7,8]. In addition, TiO_2_NPs were intensively used in many industrial processes thanks to their antimicrobial and organic catalytic features [9,10].

The cytotoxic effects induced by TiO_2_NPs are due to the large surface area to volume ratio that ensures great chemical reactivity and penetration capability in living cells. Some studies were conducted in vitro using different cell lines in order to quantify the biological effects provoked by TiO_2_NP exposure [11,12,13,14]; nevertheless, the results obtained by the golden standard assays have completely neglected the effects of these NPs on cell mechanics.

The cell generates forces and, in turn, is constantly subjected to mechanical stimuli exerted by the environment. The generation and the response to mechanical forces triggers numerous intracellular biochemical pathways that regulate different physiological cellular functions, including homeostasis, viability, stem cell differentiation, migration, mitosis, phagocytosis, endocytosis and apoptosis [15,16]. In the intracellular and external mechanical transduction signals, the cytoskeleton has a crucial role [17,18]. It is formed by a dense polymer meshwork that gives structure and shape to the cells; in addition, the cytoskeleton provides intracellular transport phenomena and the local force distribution through deformation and rearrangement of the meshwork [17]. In particular, assembly or disassembly of actin filaments induces alterations in terms of cytoskeletal tensegrity [19].

Starting from these statements, we have evaluated the effects induced by TiO_2_NP exposure on human breast adenocarcinoma cell line (MCF-7) from a mechanical point of view. By means of Atomic Force Microscopy (AFM) and Confocal Laser Scanning Microscopy (CLSM), we focused on actin rearrangement and the consequential changes in mechanical behaviour at a cellular level upon TiO_2_NP treatment at two different concentrations (25 and 50 µg/mL) and two time points (24 and 48 h).

Our approach could represent a new standard test that, combined with conventional biological studies, could help to fully understand the NPs’ cytotoxic outcomes at a cellular level.

## 2. Results

### 2.1. Characterization of TiO_2_NPs

**Figure 1:**
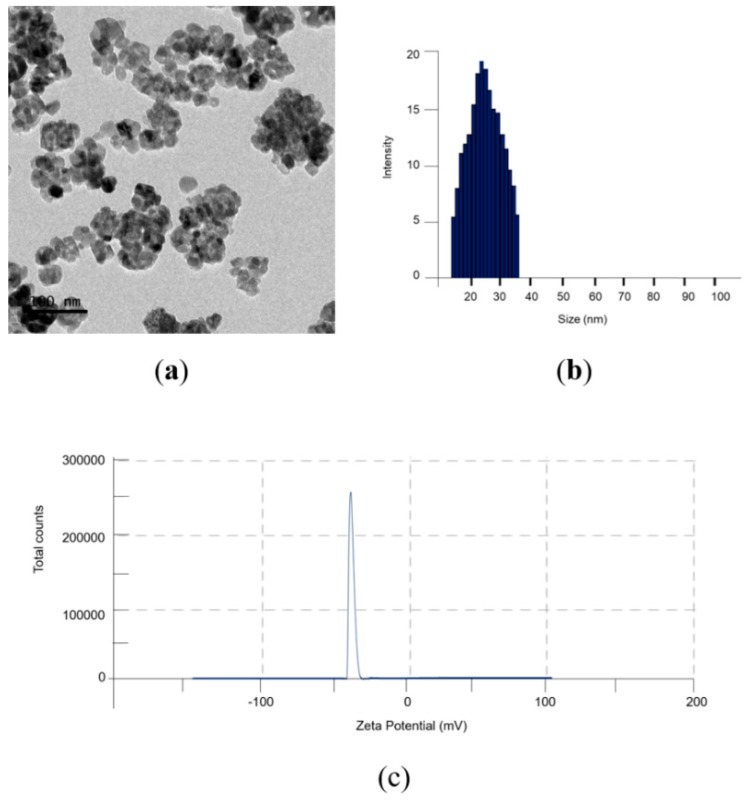
Characterization of TiO_2_ nanoparticles (NPs) in water. (**a**) Representative TEM image, (**b**) Dynamic Light Scattering (DLS) and (**c**) ζ-potential measurements.

The synthetized TiO_2_NPs were firstly characterized by means of TEM in order to analyze their morphology (Figure 1a). The TiO_2_NPs were monodispersed, having a size of (28 ± 12) nm. Monodispersion and a diameter of (33 ± 7) nm resulting from Dynamic Light Scattering (DLS) measurements (Figure 1b) were in good accordance with TEM analysis. In addition, ζ-potential measurement displayed a value of (34 ± 5) mV in water (Figure 1c).

In Dulbecco’s modified Eagle’s medium (DMEM), the size increased due to the formation of protein corona on the NPs’ surface from the serum proteins in cell culture media as previous reported [20]. In addition, the charge of the NPs changed as a function of the fetal bovine serum (FBS) concentration becoming more negative (data not shown [20]).

### 2.2. Uptake of TiO_2_NPs in MCF-7 Cell Line

**Figure 2:**
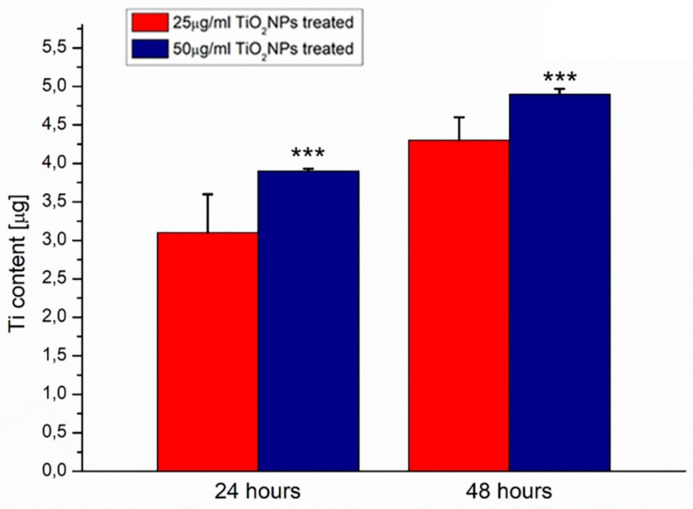
Uptake of TiO2NPs in human breast adenocarcinoma (MCF-7) cell line after 24 and 48 h of TiO_2_NP exposure at two concentrations (25 and 50 µg/mL). Data reported were calculated as average ± SD on three independent experiments, and the statistical significance respect to the control was represented (*** *p*-value < 0.005).

We performed Inductively Coupled Plasma-Atomic Emission Spectrometry (ICP-AES) elemental analysis over lysed cells in order to quantify the TiO_2_NPs internalized by cells. MCF-7 cells were treated with 20 and 50 μg/mL of NPs. The experimental data showed the uptake of NPs with a time-dependent internalization efficiency (Figure 2). Titanium (Ti) intracellular content was (3.1  ± 0.5) μg and (3.90  ±  0.03) μg after 24  h following exposure of 25 and 50 μg/mL of TiO_2_NPs, respectively. The amount of Ti was (4.3  ± 0.3) μg after 25 μg/mL of TiO_2_NPs of incubation and (4.8  ±  0.1) μg after 50 μg/mL of TiO_2_NPs after 48 h.

### 2.3. Effects of TiO_2_NPs on MCF-7 Cell’s Viability

**Figure 3:**
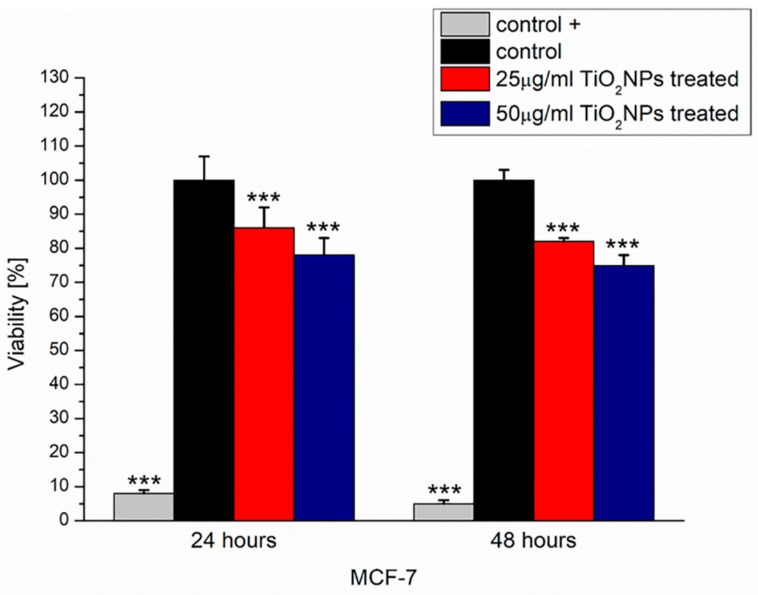
Viability assay (WST-8) on MCF-7 cells after 24 and 48 h TiO_2_NP exposure at two concentrations (25 and 50 µg/mL). Viability of TiO_2_NP-treated cells was normalized to non-treated control cells. As a positive control (control +), cells were incubated with 5% DMSO. Data reported were calculated as average ± SD on three independent experiments, and the statistical significance respect to the control was represented (*** *p*-value < 0.005).

The viability of MCF-7 exposed to TiO_2_NPs at 25 and 50 µg/mL for 24 and 48 h was evaluated by Cell Counting Kit 8 (WST-8) assay (Figure 3).

The treatment with TiO_2_NPs decreased MCF-7 viability in a concentration-dependent manner: indeed, after 24 h of exposure, the percentage of viability with respect to the untreated control was reduced by about 14% in the case of 25 µg/mL of concentration and about 22% in the case of 50 µg/mL. This trend was confirmed after 48 h of TiO_2_NP exposure: the percentage of reduction in cell viability became equal to 18% in the case of 25 µg/mL of concentration and equal to 25% in the case of 50 µg/mL.

**Figure 4:**
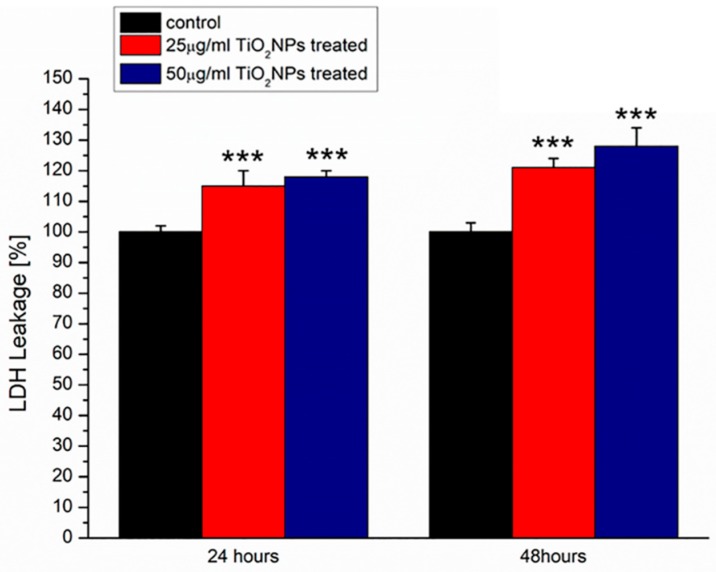
Lactate dehydrogenase (LDH) assay on MCF-7 cells incubated with 25 and 50 µg/mL of TiO_2_NPs at 24 and 48 h. Percentage of LDH leakage of nanoparticle-treated cells are expressed relative to non-treated control cells. Positive controls (P) consisted of the treatment of cells with 0.9% Triton X-100 showing ca. 500% LDH increase (not shown). Data are reported as mean ± SD from three independent experiments; the statistical significance respect to the control (*n* = 8) was represented (*** *p*-value < 0.005).

The cell membrane damage was analyzed following the lactate dehydrogenase (LDH) release, a cytosolic enzyme. LDH leakage assay was in close agreement with viability results: the cell membrane disruption induced an increase of LDH release that was dose and time dependent (Figure 4). In particular, at 48 h using 25 µg/mL of TiO_2_NPs, the value was (121 ± 3)% whereas at 50 µg/mL of TiO_2_NPs, the LDH release was (128 ± 6)% with respect to the negative control (100%).

### 2.4. Confocal Studies

**Figure 5:**
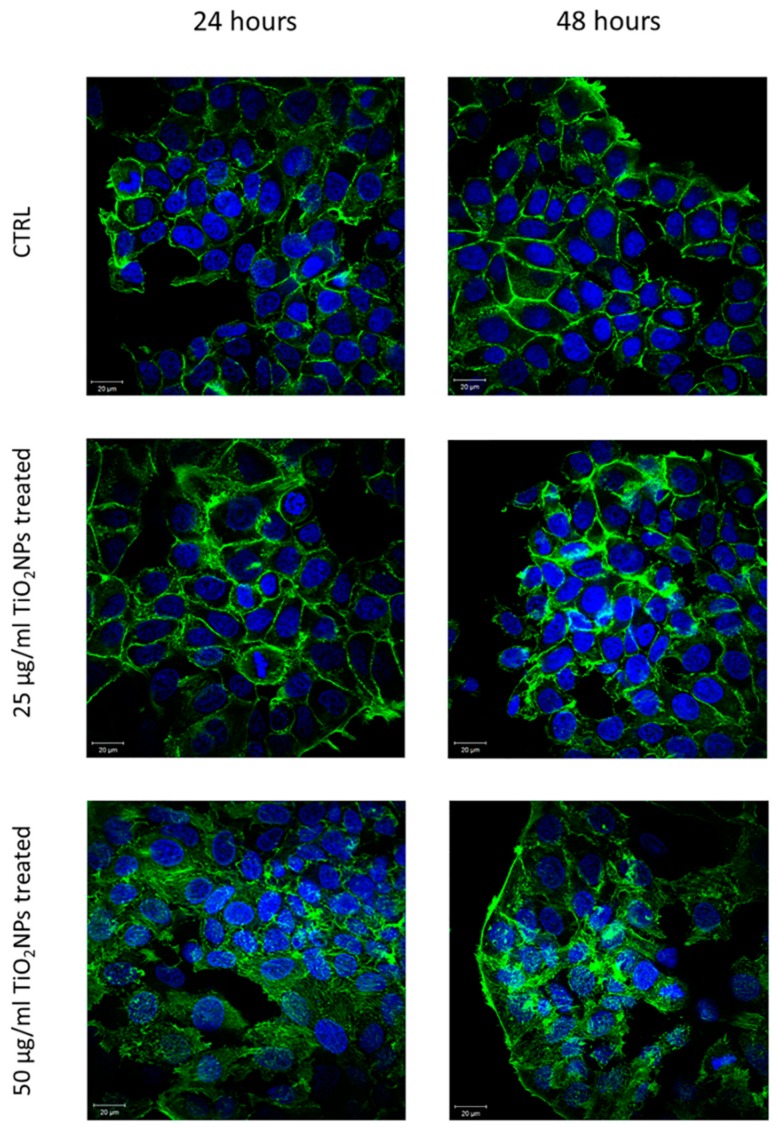
Representative confocal acquisitions on MCF-7 cells control and treated with 25 and 50 µg/mL TiO_2_NPs, at two different exposure times: 24 and 48 h. Cells fixed were labelled with DAPI (D9542, Sigma Aldrich) and phalloidin-FITC (P5282, Sigma Aldrich). The images were acquired by a Zeiss LSM700 (Zeiss, Germany) confocal microscope implemented on Axio Observer Z1 (Zeiss, Germany) inverted microscope using an oil immersion objective (×100, 1.46 NA). All data were processed by ZEN software (Zeiss, Germany).

The effects of TiO_2_NP exposure on MCF-7 cells in terms of actin fibers reorganization and nuclear morphological alterations were investigated by CLSM (Figure 5).

**Figure 6:**
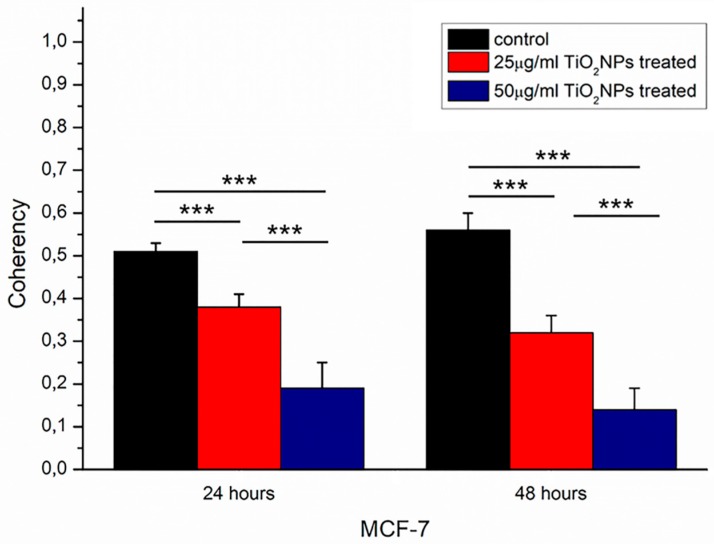
Histogram reports coherency values, expressed as mean and their respective standard deviation, calculated on 15 cells for each sample by means OrientationJ plugin (ImageJ software) on confocal acquisitions. Results were compared by *t* test to the control value, and the statistical significance was represented (*** *p*-value < 0.005).

After 48 h, the MCF-7 control exhibited a typical epithelial shape showing well-defined actin architecture: F-actin filaments were organized into higher-order structures, forming bundles. In treated samples, cells exhibited a loss of adherent adhesions and a less orderly actin network. This observation was confirmed by means of a coherency quantification parameter (Figure 6). In detail, after 24 h its value was (0.42 ± 0.03) for the control sample, and it changed to (0.34 ± 0.03) after 25 µg/mL of TiO_2_NP exposure; after 50 µg/mL of TiO_2_NP exposure, the value became (0.16 ± 0.06).

After 48 h, the coherency value of the MCF-7 control was (0.56 ± 0.04), an increase with respect to the corresponding sample at 24 h. The TiO_2_NPs’ incubation up to 48 h induced a reduction of coherency, that was (0.28 ± 0.04) and (0.13 ± 0.05) for TiO_2_NPs at a concentration of 25 and 50 µg/mL, respectively.

**Figure 7:**
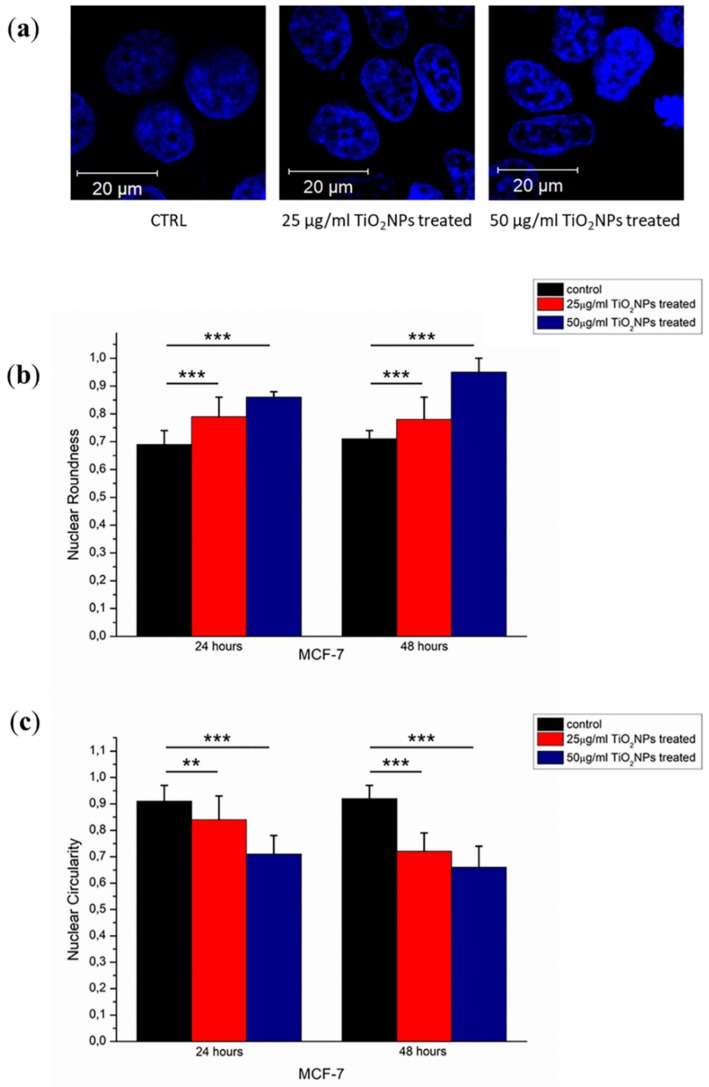
Representative confocal images of nuclei (**a**)Histograms reported the mean values and their respective standard deviation of nuclei circularity (**b**) and roundness (**c**). The statistical significance of results respect to control cells was evaluated by *t* test, and reported in histograms (** *p* < 0.01 and *** *p* < 0.005.).

The nuclear morphological perturbations (Figure 7a) were evaluated in terms of circularity (Figure 7b) and roundness (Figure 7c). The TiO_2_NPs induced a statistically significant reduction of circularity only after 48 h of TiO_2_NP exposure. Specifically, the circularity value at 24 h was equal to (0.93 ± 0.04) for control cells and it become (0.86 ± 0.06) and (0.85 ± 0.07) after 25 and 50 µg/mL of TiO_2_NPs treatment, respectively.

After 48 h, the circularity result was (0.96 ± 0.04) for the control sample, whereas the value was reduced to (0.77 ± 0.09) for TiO_2_NPs at 25 µg/mL and (0.72 ± 0.08) after 50 µg/mL of TiO_2_NPs treatment.

The roundness parameter increased after 24 h of TiO_2_NPs exposure; this value was (0.62 ± 0.07) for the control that changed to (0.78 ± 0.08) and (0.83 ± 0.04) for 25 and 50 µg/mL TiO_2_NPs, respectively. Furthermore, this trend was more evident after 48 h of exposure: roundness of MCF-7 control (0.68 ± 0.03) turned into (0.78 ± 0.09) and (0.98 ± 0.02) for 25 and 50 µg/mL of TiO_2_NPs.

### 2.5. Mechanical Investigations

Elastic behavior of the cellular membrane was evaluated in terms of Young’s Modulus (E) parameter. It was quantified analyzing the force-distance curves extracted in correspondence of the nuclear region and cytoplasmic areas, as described in the Materials and Method section.

**Figure 8:**
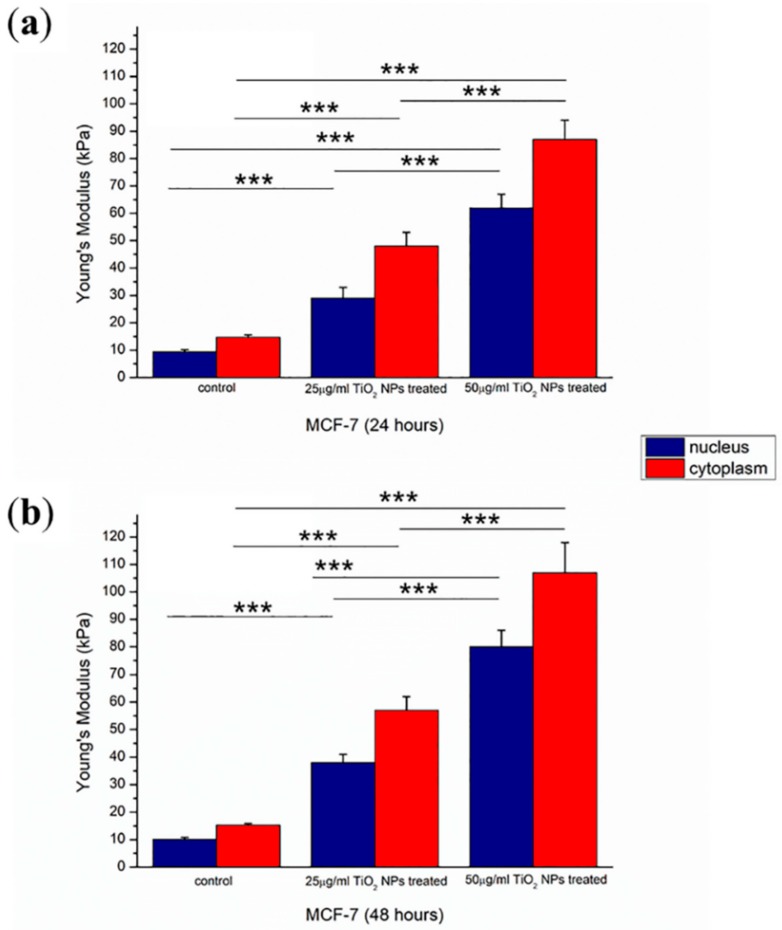
Histograms reported Young’s Modulus (E) of MCF-7 control and MCF-7 exposed to TiO_2_NPs at two different concentrations (25 and 50 µg/mL) for 24 h (**a**) and 48 h (**b**). E values were expressed as mean ± SD. The statistical significance of results, respect to control, was showed (*** *p* < 0.005).

After incubation of TiO_2_NPs, E value increased in a dose dependent manner: at 24 h (Figure 8a), Young’s Modulus calculated in correspondence of nuclear region ranged from (9.4 ± 0.8) kPa to (29 ± 4) kPa for 25 µg/mL of TiO_2_NPs and to (62 ± 5) kPa for 50 µg/mL of TiO_2_NPs. In correspondence of cytoplasmic area, elasticity value in the control sample (14.7 ± 0.9) kPa changed to (48 ± 5) kPa and (87 ± 7) kPa after TiO_2_NP exposure with 25 and 50 µg/mL concentrations, respectively.

After 48 h of exposure (Figure 8b), the effect of the Young’s Modulus increased, becoming more evident. In fact, its value ranged from (10.1 ± 0.7) kPa to (38 ± 3) kPa and (80 ± 6) kPa in the case of the control, 25 µg/mL TiO_2_NPs and 50 µg/mL TiO_2_NPs, respectively, in correspondence to nuclear area. Young’s Modulus calculated in correspondence of nuclear region turned from (9.4 ± 0.8) kPa to (29 ± 4) kPa for 25 µg/mL of TiO_2_NPs and (62 ± 5) kPa for the higher concentration. In correspondence of cytoplasmic area, the elasticity value in the control sample (14.7 ± 0.9) kPa changed to (48 ± 5) kPa and (87 ± 7) kPa, after 25 and 50 µg/mL of TiO_2_NP exposure, respectively.

## 3. Discussion

The rapid growth of ENPs applications in many fields has generated an increased apprehension about the potential adverse effects on living organisms and the environment. Although several in vitro and in vivo studies were conducted to investigate the alterations in biological systems, many gaps need to be filled regarding the cellular mechanisms induced by interaction between NPs and living organisms.

The exact mechanism of TiO_2_NP toxicity is still under investigation. Lozano et al. [21] observed the formation of small aggregates of TiO_2_NPs that facilitate the interaction with cells. De Matteis et al. [22] hypothesized a possible uptake route through a NPs ionization that was dependent on crystalline form and UV irradiation which facilitate the internalization across the plasma membrane.

Moschini et al. [23] reported that TiO_2_NPs were internalized by cells through the endocytic pathway showing that, only after 24 h, epithelial cells lines underwent a morphology alteration. TiO_2_ toxicity was correlated to oxidative stress and subsequent NF-κB signaling pathways’ activation: this mechanism is typical of metal nanoparticles that induced reactive oxygen species (ROS) formation by Fenton-like reactions and mitochondrial injury [24]. In addition, the ROS production triggered cell death by lipid peroxidation, inducing cell membrane disruption. The in vitro studies suggested the ability of TiO_2_NPs to migrate in the bloodstream after phagocytosis by macrophages binding proteins [25]. Binging with plasma proteins can be associated with the negative surface charge that was suitable to interact with amino acids containing –OH, –NH, and –NH_2_ in their chains [26]. A deeper understanding of biophysical response due to NPs’ interaction requires the analysis of cytotoxicity from a biomechanical point of view, because it has been demonstrated how biological and physiological events change cell mechanics and vice versa [27,28,29,30].

These considerations have led to the study of cortical cytoskeleton remodeling and the consequent elastic behavior alteration of cellular membrane in MCF-7 cell line, after TiO_2_NP exposure.

The reduction of the cell viability induced by TiO_2_NPs was dose and time dependent, and the same trend was observed after membrane damage evaluation; but these results were not sufficient to suggest the level of seriousness associated with TiO_2_NP exposure. There are additional indications about the relevance of induced cytotoxic effects by means of morphomechanical quantifications. In histopathological studies, nuclear shape alteration is a qualitative distinct characteristic of cancer progression [31,32]. Keeping this in mind, it is conceivable to link the variation of nuclear morphological parameters to cellular physiological state [33]. The chromatin remodeling induces an alteration of nuclear morphology that could be quantified in terms of roundness and circularity: in our case, the exposure to TiO_2_NPs provoked an increase of roundness and circularity value reduction, indicating a pre-apoptotic condition [34]. In addition, the cytoskeletal network of MCF-7 treated with TiO_2_NPs was partially destroyed, influencing cellular adhesion [35]. In detail, the treatment damaged cell–cell adhesions and each treated cell appeared less strictly connected with neighboring cells. The alteration in cellular adherent junctions entails modification in metabolic processes, protein synthesis and viability [36].

Our results were in accordance with evidence presented by Setyawati and coworkers [37], that showed how TiO_2_NPs triggered a detrimental impact on adherent junctions in human lung microvascular endothelial cells (HMVEC). The cell–cell adhesions in MCF-7 cells appeared damaged also after Selenium NPs (SeNPs), Silver NPs (AgNPs) and commercial TiO_2_NP exposure [22,38,39].

Furthermore, F-actin filaments lost their proper organization assuming a more disordered configuration. This effect became more evident after 48 h of exposure; in fact, the coherency parameter decreased by 50% for 25 µg/mL and 77% for 50 µg/mL concentrations of TiO_2_NPs.

A lower degree of fiber orientation associated with whole cytoskeletal architecture reorganization involves alteration of cell mechanical properties.

The quantification of elastic behavior, in terms of Young’s Modulus, represents an advantageous biological marker to characterize physiological cellular health. Among all techniques used to evaluate elastic parameter, AFM is the preferred tool in the biological field, and it has been widely used to assess mechanical properties of several cell types in order to understand the existent link between mechanical properties and biological phenomena [40,41]. In this work, AFM was used, in force volume mode, to quantify the alteration in cellular membrane elasticity, after TiO_2_NPs interaction.

For both concentrations used and for both time points, the effect of TiO_2_NP exposure was very strong on MCF-7 cell elasticity. After 24 h, TiO_2_NPs caused the increase of stiffness of the cellular membrane in correspondence of nuclear area and cytoplasmic areas. This effect became highlighted when the time of exposure was prolonged for 48 h.

The found reduction of elastic behavior in the cellular membrane upon TiO_2_NP interaction has also been demonstrated in human adenocarcinoma alveolar basal epithelial (A549) cell line, whereas Young’s modulus decreased. In human epithelial colorectal adenocarcinoma (Caco-2) cell line subjected to same treatment, the effect was similar [20].

In the light of the above, we conclude that the morphomechanical response of different cell lines depends on the physico-chemical properties of NPs and how they interact with the cellular membrane.

For this reason, it is clear that to gain an accurate understanding of cytotoxic outcomes induced by ENPs exposure, the supplementation of biological investigations with biomechanical characterizations is needed. This approach appears to be a prospective method for standardizing NPs toxicity assessment investigations.

## 4. Materials and Methods

### 4.1. Synthesis and Characterization of TiO_2_NPs

TiO_2_NPs were prepared following the sol-gel method [42] with some modifications [20]. Briefly, Titanium (IV) isopropoxide (TTIP, 99,9%, 377996, Sigma Aldrich, Darmstadt, Germania) was dropped in a solution of ethanol and milliQ water (5:1:1) under stirring in acidic conditions (pH 3). NPs were incubated for 5 h at 30 °C first, and then at 430 °C for 3 h to obtain a white nano powder.

Synthetized TiO_2_NPs were characterized in transmitted electron microscopy: carbon-coated copper grids (Formvar/Carbon 300 Mesh Cu) were used as a substrate on which a dilute solution of TiO_2_NPs in water was dropped, then samples were measured using a JEOL Jem 1011 microscope (JEOL Inc, Peabody, MA, USA), operating at an accelerating voltage of 100 kV.

In addition, TiO_2_NPs were measured by Dynamic Light Scattering (DLS, ZEN3600, Malvern Instruments Ltd., Malvern, UK) and ζ-potential using a Zetasizer Nano-ZS (Malvern, UK) at 25 °C in aqueous solutions (pH 7).

### 4.2. Cell Culture

Human breast cancer cell line (MCF-7) was purchased from the American Type Culture Collection (ATCC^®^ HTB-22™). Cells were grown in Dulbecco’s modified Eagle’s medium (DMEM, D5796, Sigma Aldrich, Darmstadt, Germania), supplemented with 10% (*v*/*v*) fetal bovine serum (FBS, 12107C), 1% (*v*/*v*) l-glutamine (G7513), 1% (*v*/*v*) penicillin/streptomycin (P4333). Cells were maintained at 37 °C in a humidified atmosphere of 5% CO_2_ (*v*/*v*). All reagents were purchased by Sigma Aldrich (Darmstadt, Germania)

### 4.3. Determination of the Intracellular Uptake of TiO_2_NPs with Elemental Analysis

First, 10^5^ MCF-7 cells were seeded in 1 mL of medium in a six well plate. After 24 h at 37 °C, the medium was replaced with fresh DMEM containing the TiO_2_NPs (25 and 50 µg/mL). After 24 and 48 h of exposure at 37 °C, DMEM was removed and the cells washed several times with Phosphate Buffered Saline (PBS) (pH 7.4). Cells were trypsinized and counted using an automatic cell counting chamber. Next, 360,000 cells were suspended in 200 µL of milliQ, treated with HCl/HNO_3_ 3:1 (*v*/*v*) and diluted to 5 mL: the obtained solution was analyzed to evaluate Ti content. Elemental analysis was carried out by Inductively Coupled Plasma-Atomic Emission Spectrometry (ICP-AES) with a Varian Vista AX spectrometer.

### 4.4. WST-8 Assay and LDH Assay

MCF-7 cells were seeded in 96 well microplates at a concentration of 5 × 10^3^ cells/well and stabilized in an incubator for 24 h. Successively, TiO_2_NPs at two different concentrations (25 and 50 µg/mL) were added to cell media. After incubation times of 24 and 48 h, standard WST-8 assay (96992, Sigma Aldrich, Darmstadt, Germania) and the lactate dehydrogenase (LDH) leakage assay, using the CytoTox-ONE Homogeneous Membrane Integrity Assay reagent (G7890, Promega, Madison, WI, USA), were performed to conduct viability testing and to evaluate the membrane damage, respectively.

The WST-8 assay procedure that was used is described in our previous work [43].

The amount of lactate dehydrogenase (LDH), a soluble cytosolic enzyme released after cell lysis, was measured by reading absorbance at 490 nm using a Bio-Rad microplate spectrophotometer. The increase of the LDH activity in culture supernatant is proportional to the number of cells lysed. Data were expressed as mean ±SD. Mean values differences between cells treated and respective controls were considered statistically significant performing a t-student test (*p*-value < 0.05)

### 4.5. Confocal Laser Scanning Microscopy

#### 4.5.1. Preparation of Samples

MCF-7 cells were seeded at a concentration of 8 × 10^4^ cells/mL in glass Petri dishes (Sarstedt, Germany). After 24 h of stabilization, the culture media was supplemented with TiO_2_NPs at two different concentrations: 25 and 50 µg/mL. After TiO_2_NP exposure for 24 and 48 h, the medium was removed then three washes with Phosphate Buffered Saline (PBS, D1408, Sigma Aldrich) were performed.

Samples were fixed by using glutaraldehyde (G5882, Sigma Aldrich) at 0.25% in PBS for 10 min. After two washes with PBS, Triton X-100 (Sigma Aldrich) at 0.1% for 5 min was used to permeabilize the cell membrane of fixed cells before staining the nuclei and actin cytoskeleton. In detail, 1 μg/mL of DAPI (D9542, Sigma Aldrich) for 5 min and 1 µg/mL of phalloidin-FITC (P5282, Sigma Aldrich) for 1 h were used to label nucleic acid and F-actin, respectively.

#### 4.5.2. Confocal Experiments

Acquisitions were performed by Zeiss LSM700 (Zeiss, Germany) confocal laser scanning mounted on Axio Observer Z1 (Zeiss, Germany) inverted microscope, using the Alpha Plan-Apochromat (Zeiss, Germany) 100× oil-immersion objective with 1.46 NA. The fluorescent images were obtained exciting fluorescent dyes by means laser radiations having wavelength at 405 nm and 555 nm for DAPI and phalloidin-FITC, respectively.

The confocal images were acquired on the middle and cortical focal plane and successive acquisitions performed for each sample (control, 25 and 50 µg/mL TiO_2_NPs at 24 and 48 h) were analyzed by ImageJ 1.47v software (National Institutes of Health, Bethesda, MD, USA) by using specific software tools.

In detail, the nuclear morphology was quantified in terms of two shape descriptor parameters: circularity and roundness. Circularity parameter compares an object to a circle, it is ranges from 0 to 1 (for a perfect circle). The Roundness parameter has a similar definition to circularity, but it does not depend on local irregularity of an object surface.

The cytoskeletal actin organization was characterized in terms of coherency by means of OrientationJ plugin (ImageJ software); this parameter indicates the local orientation of actin filaments. In detail, the value of coherency ranges from 0 (isotropic orientation) to 1 (perfectly oriented structures) [44].

All results were obtained as means calculated on 15 cells, and the difference in mean after a treatment on a sample in comparison to before was statistically analyzed by means of a paired two-tailed *t*-test. The statistical difference of results was considered significant for *p*-value < 0.05.

### 4.6. Atomic Force Microscopy

#### 4.6.1. Preparation of Samples

MCF-7 cells were seeded in plastic Petri dishes (Corning) at a concentration of 8 × 10^4^ cells/mL and stabilized for 24 h. Subsequently, TiO_2_NPs, at concentrations of 25 and 50 µg/mL, were added to the culture medium, then the cells were incubated for 24 and 48 h. After exposure time, the medium with NPs was removed and washed three times using PBS. Glutaraldehyde 0.25% was used to fixed samples. After 20 min of Glutaraldehyde treatment, cells were washed with PBS.

#### 4.6.2. AFM Experiments

Indentation force curves were recorded in Force Volume (FV) mode by using an advanced scanning probe microscope (Bioscope Catalyst, Bruker Inc., CA, USA) set up on an inverted optical microscope (Zeiss Observer Z1, Zeiss, Germany). Acquisitions were performed using a Silicon Nitride V-shaped Bruker’s Sharp Microlever (MSNL, tip C), having a nominal spring constant of 0.01 N/m. For the first of sample measurements, thermal tune method [45,46] was used to calibrate with high precision the cantilever spring constant. FV acquisitions were recorded in area of 50 µm × 50 µm (Scan Area) with a resolution of 512 (Number of sample) × 128 (Sample per line) × 128 (Lines). In addition, Ramp Rate was set equal to 4.88 Hz, FV scan rate to 0.03 Hz, and Trigger Threshold to 50 nm, therefore the maximum interaction force between the AFM tip and the cell surface amounted to 100 pN. More to the point, operating in FV mode, we simultaneously acquired topography and tip-sample interaction data on a specific area; in this way a direct correspondence between a single point in a topography map and a single indentation curve was possible, then we manually selected the 25 curves that corresponded to the nuclear compartment and the cytoplasmic one. This procedure was performed on 20 cells for each sample (control, 25 µg/mL, 50 µg/mL). By using the Nanoscope Analysis software (Bruker Inc., CA, USA), the local Young’s Modulus (E) was quantified for all curves acquired and the elasticity of the nuclear/cytoplasmic region was calculated as the average on single curves selected from the region of interest, using the procedure described in our previous work [47,48].

The elasticity data were analyzed and graphed using the OriginPro software (OriginLab version 8, MA, USA). The statistical significance of results was established by means of a paired two-tailed *t*-test: the differences were considered statistically significant for *p*-values < 0.05.

## 5. Conclusions

In this work, we show how the cytotoxic effects induced by TiO_2_NPs on MCF-7 cells are underestimated if the viability test is only considered. The TiO_2_NP exposure leads to meaningful change in Young’s Modulus, suggesting a strong alteration of cell physiology. Alterations of elastic behavior is due to a significant rearrangement of F-actin fibers and a remodeling of the nuclear compartment. In this scenario, although cell viability is not strongly compromised, the massive change in morphomechanical properties, mainly in cellular membrane, suggests important alterations in cell physiology that could induce the onset of many diseases, such as cancer.
